# Circ_0058106 promotes proliferation, metastasis and EMT process by regulating Wnt2b/β-catenin/c-Myc pathway through miR-185-3p in hypopharyngeal squamous cell carcinoma

**DOI:** 10.1038/s41419-021-04346-8

**Published:** 2021-11-09

**Authors:** Ce Li, Wenming Li, Shengda Cao, Jianing Xu, Ye Qian, Xinliang Pan, Dapeng Lei, Dongmin Wei

**Affiliations:** Department of Otorhinolaryngology, Qilu Hospital, Shandong University; NHC Key Laboratory of Otorhinolaryngology (Shandong University), 107 West Wenhua Road, 250012 Jinan, Shandong China

**Keywords:** Head and neck cancer, Head and neck cancer

## Abstract

Hypopharyngeal squamous cell carcinoma (HSCC) accounts 95% of hypopharyngeal cancer, which is characterized by high early metastasis rate and poor prognosis. It is reported that circular RNA is involved in the occurrence and development of cancer; however, the role of circRNA in hypopharyngeal cancer has little been investigated. We performed hypopharyngeal carcinoma circRNA microarray and qRT-PCR verification. The results showed circ_0058106 expression level was significantly upregulated in tumor tissues than in corresponding normal tissues. We found that circ_0058106 upregulation promoted proliferation, migration and invasion of HSCC cells, while knockdown of circ_0058106 inhibited proliferation, migration and invasion of HSCC cells both in vitro and in vivo. Bioinformatics predicted circ_0058106 may interact with miR-185-3p. We verified circ_0058106 directly bound miR-185-3p and downregulated miR-185-3p expression by using dual-luciferase reporter assay and qRT-PCR. Moreover, we proved circ_0058106 promoted HSCC cells tumorigenesis and EMT process by regulating Wnt2b/β-catenin/c-Myc pathway via miR-185-3p. In conclusion, our findings firstly confirmed the carcinogenic effect of circ_0058106 in promoting HSCC cells tumorigenesis, metastasis, invasion and EMT process by regulating Wnt2b/β-catenin/c-Myc pathway through sponging miR-185-3p, indicating that circ_0058106 may be a new therapeutic target and prognostic marker for HSCC.

## Introduction

Hypopharyngeal cancer is a malignant tumor derived from the epithelial tissue of the laryngeal mucosa, accounts for about 3% of head and neck malignancies [1]. The pathological type of hypopharyngeal carcinoma is mainly squamous cell carcinoma [2]. Although the incidence of hypopharyngeal cancer is relatively low, its occurrence is relatively hidden and hard to detect. Therefore, its prognosis is poor and mortality rate is high [3]. Most hypopharyngeal cancers have spread to regional lymph nodes or have metastases when they are first diagnosed. The 5-year overall survival rate of hypopharyngeal cancer is only 30-35% [4]. The treatment methods of hypopharyngeal cancer is mainly comprehensive treatment methods containing surgery combine with radiotherapy and chemotherapy [5]. However, survival rates of Hypopharyngeal squamous cell carcinoma (HSCC) patients have not markedly improved, new therapeutic method and target of HSCC are urgently needed.

Circular RNA (circRNA) is a kind of special noncoding RNA (noncoding RNA, ncRNA). Most of them are produced by alternative splicing of the precursor mRNA of the protein-encoding gene [6]. The 5′ and 3′ ends of circRNA are directly connected to form a circular closed structure, which cannot be degraded by endonucleases and is more stable than linear RNA [7]. CircRNA plays an important role in tumor development, metastasis, invasion, and drug resistance, and is expected to become a new molecular marker and therapeutic target for tumor diagnosis and therapy [8]. Several circRNAs have been reported to regulate HSCC tumorigenesis. For example, circMATR3 is upregulated in HSCC tissues and promotes the proliferation, migration and invasion of HSCC cells [9]. circMORC3 is downregulated in HSCC tissue and plasma and may be a potential biomarker for early diagnosis of HSCC [10]. However, information about functions and mechanisms of circRNA in HSCC is limited.

In this study, we studied the functions and molecular mechanisms of circ_0058106 in the occurrence and development of HSCC. Our results suggest that circ_0058106 could promote HSCC proliferation, migration, invasion and EMT process by regulating Wnt2b/β-catenin/c-Myc pathway via direct binding to miR-185-3p. The results of this study reveal potential new molecular target and new mechanisms for the development of hypopharyngeal cancer. Moreover, circ_0058106 may be a new prognostic and metastasis biomarker of hypopharyngeal carcinoma.

## Materials and methods

### Cell lines and cell culture

Fadu, TU686, TU212 and Detroit562 cells were obtained from American Type Culture Collection and identified by STR profiling. Cells were cultured in DMEM high glucose (BasalMedia, Shanghai, China) containing 10% fetal bovine serum (Sigma, Saint Louis, USA). Phosphate Buffer Saline (BasalMedia, Shanghai, China) and Tyrisin (Macgene, Beijing, China) were used for cell passage cultivation. Serum-Free Cell Freezing Medium (New cell& molecular Biotech, Suzhou, China) was used for cell cryopreservation.

### Expression plasmids and transfection reagents

Circ_0058106 (WT)-pmirGLO, circ_0058106 (MUT)-pmirGLO, Wnt2b (WT)-pmirGLO, Wnt2b (MUT)-pmirGLO plasmids and miR-185-3p mimics were constructed by Biosune Technologies (Shanghai, China). Lip3000 (Invitrogen, Carlsbad, USA) and Opti-MEM (Gibco, Carlsbad, USA) were used for plasmid transfection.

### Antibodies

The following antibodies were used for western blot: c-Myc (Abcam, ab32072, 1:1000), Wnt2b (Abcam, ab178418, 1:1000), beta Catenin (Abcam, ab68183, 1:1000), E-cadherin (Abcam, ab40772, 1:1000), α-catenin (Abcam, ab51032, 1:1000), N-cadherin (Abcam, ab76011, 1:1000), Vimentin (Abcam, ab92547, 1:1000), β-actin (Abcam, ab8226, 1:1000), Goat anti mouse second antibody (Cell Signaling Technology, 5470S, 1:15000), Goat anti rabbit second antibody (Cell Signaling Technology, 5151S, 1:30000).

### Establish circ_0058106 stably overexpressing and knockdown Fadu cell lines

Circ_0058106-OE or LV5 control plasmid, as well as circ_0058106-sh or LV3 control plasmid was transfected into Fadu cells by using Lentivirus packaging systems (GenePharma, Shanghai, China). Transfection efficiency was verified by using immunofluorescence. After transfection, circ_0058106 expression levels were verified by using qRT-PCR.

### Quantitative real-time PCR

Total RNA was extracted using TRIzol Reagent (Sigma, Saint Louis, USA) and then transcribed into cDNA using Reverse Transcription System (Accurate Biotechnology, Shanghai, China) with a random primer. SYBR Green Mastermix (Takara, Kyoto, Japan) and 7900HT Fast Real-Time PCR System (ABI, Waltham, USA) were used for quantitative real-time PCR. GAPDH mRNA or U6 was served as a normalization. All oligonucleotide 5′-GCCTGGGAGCAAGTCTACAG-3′ and 5′-ACAGGGGAACTTGAAGGCAG-3′ for circ_0058106 (convergent). 5′-ATCCAAGCGGAGAGAGTCAG-3′ and 5′- AACTCCAGGTTTAAGGCCGC-3′ for circ_0058106 (divergent). 5′-GTCTCCTCTGACTTCAACAGCG-3′ and 5′-ACCACCCTGTT GCTGTAGCCAA-3′ for GAPDH. 5′-TCATCAGCAGGGGTAGTCCA-3′ and 5′-CGGACACCGTAGTGGATGTT-3′ for Wnt2b. 5′-GGGTCTCCTCCCAGAGAAGT-3′ and 5′-GTTGGGGAAGCTCGTCTGTC-3′for FN1. 5′-GTAATCTTTAGGGGCTGGCTTT-3′ and 5′-TATGCTTGTTCTCGTCTCTGTGTC-3′ for miR-185-3p. 5′-CAGCACATATACTAAAATTGGAACG-3′ and 5′- ACGAATTTGCGTGTCATCC-3′for U6.

### Western blot

For western blotting, used RIPA lysis buffer supplemented with protease inhibitors to prepare whole cell lysates. The protein was separated by gel electrophoresis and transferred to a PVDF membrane (Millipore, Billerica, USA), then blocked by 5% BSA (Beyotime Biotechnology, Shanghai, China). Put the membrane in blocking buffer containing primary antibody overnight at 4 °C, then washed in TBST (Beyotime Biotechnology, Shanghai, China), and incubated in TBST containing secondary antibody. Keep at room temperature for 1 h. After washing again in TBST, detected proteins by using Odyssey infrared fluorescence scan imager (Li-COR, Lincoln, USA).

### Wound healing assay

Fadu cells were cultured in a 6-well plate, when reached 100% confluence, used a 200 μL pipette tip to form stripes on the monolayer and cultured in serum-free DMEM medium for 24 h. Images of the cells were captured at 0 and 24 h after form stripes.

### Transwell migration assay and transwell invasion assay

Transwell porous polycarbonate membrane insert (Corning, New York, USA) was used for migration measurement, and Matrigel-coated invasion chamber (Corning, New York, USA) was used for invasion measurement. For transwell migration assay, 3 × 10^4^ cells were seeded into the upper chamber and cultured in serum-free medium. For transwell invasion assay, 10^5^ cells were seeded into the upper Matrigel-coated invasion chamber and cultured in serum-free medium. The lower chamber contained medium supplemented with 20% FBS. After 24 h, cells adhered to the lower surface were fixed with methanol for 30 min and stained with crystal violet for 30 min. Then the cells on the upper membrane were removed and the cells adhered to the lower surface were washed with PBS. Captured images of stained cells by using a microscope.

### Cell viability assay

3 × 10^3^ Fadu cells were seeded in 96-well plates and cultured for 0, 24, 48, and 72 h. 10 μL CCK-8 solution (Med Chem Express, New Jersey, USA) was added to each well and incubated at 37 °C for 1 hour. Then measured the absorbance at 450 nm.

### Colony formation assay

Colony formation assay was performed to assess Fadu cells proliferation. Digested cells in the logarithmic growth phase and inoculated 300 cells (for Fadu) or 500 cells (for TU212) in each 6-well plate, cultured in DMEM medium with 10% FBS for 2 weeks; aspirated the supernatant and washed 3 times by using PBS; fixed with methanol for 30 min and stained with crystal violet for 30 min. Recorded the number of more than 30 cells as clones, counted the number of clones under a microscope and performed statistical analysis.

### EdU assay

The EdU assay was used to detect cell proliferation. 1 × 10^4^ circ_0058106-OE, LV5, circ_0058106-sh and LV3 Fadu cells were seeded on 96-well plates, and cultured in a medium containing 10% fetal bovine serum at 37 °C and 5% CO_2_ for 48 h. The detection was performed using the Cell-Light EdU-594 in vitro imaging kit (RiboBio, Guangzhou, China) according to the manufacturer’s protocol. Used a fluorescence microscope to capture the image. The results were analyzed by two blinded observers.

### Dual-luciferase reporter assay

After sequence comparison between circ_0058106 (or Wnt2b) and miR-185-3p, the fluorescein-labeled reporter gene was detected using the dual-luciferase detection system kit (Promega, Madison, USA). Fadu cells were seeded on a 48-well plate, cultured for 24 h, then co-transfected with wild-type or mutant pmiRGlo-circ_0058106 (or pmiRGlo-Wnt2b) dual-luciferase reporter vectors incorporating miR-185-3p binding sites vectors and miR-185-3p mimics or negative control by using transfection reagents. After 48 h, added passive lysis buffer (20 μL) and Luciferase Assay Reagent II (100 μL) to each well, measured the absorbance at 580 nm using a microplate reader. Then added Stop&Glo^®^ Reagent (100 μL) to the same well and measure the absorbance at 460 nm.

### Fluorescence in situ hybridization (FISH)

Circ_0058106 FISH probe labeled with 5′-CY3 and miR-185-3p probe labeled with 5′-FAM were designed by Biosune Technologies (Shanghai, China) and used in the experiment. Then performed RNA Fish assay (Genepharma, Shanghai, China) according to the manufacturer’s protocol.

### Cytoplasm and nuclear localization

The cytoplasmic and nuclear RNA purification kit (Norgen biotek, Toronto, Canada) was used to separate the cytoplasmic and nuclear fractions from the cells according to the manufacturer’s protocol. The cytoplasmic and nuclear RNA was then converted into cDNA, which was detected and analyzed by qRT-PCR.

### In vivo tumorigenesis model

To determine tumorigenesis in vivo, the mice were randomly divided into LV5 and circ_0058106-OE group, each group contained 5 mice. LV5 and circ_0058106-OE Fadu cells were cultured and cells in the logarithmic phase were digested and resuspended in PBS. The cell density was adjusted to 5 × 10^7^/ml, and 0.1 ml was injected into 6-week-old female nude mice on the armpit. The tumor size was measured with a caliper every week, and the tumor volume was determined by a standard formula: L×W^2^×0.52, where L is the longest diameter and W is the shortest diameter. After about 4 weeks, the tumors were removed and the weight and volume of the tumors were determined. The Institutional Animal Care and Use Committee (IACUC) is from the Ethics Committee of Qilu Hospital of Shandong University.

### In vivo metastasis model

For determine metastasis in vivo, the mice were randomly divided into LV5 and circ_0058106-OE group, each group contained 5 mice. LV5 and circ_0058106-OE Fadu cells were cultured and cells in the logarithmic phase were digested and resuspended in PBS. The cell density was adjusted to 5 × 10^6^/ml, and 0.1 ml was injected into 6-week-old female nude mice on the lateral tail vein. After 6 weeks, the mice were sacrificed and lung tissues were collected for HE staining.

### IHC and scoring

The procedures of IHC studies were performed as previously described [11]. In short, the sample was fixed with 10% formalin, embedded in paraffin, and sliced into 5-μm sections. Incubated the slide with primary antibody. Staining was observed in five randomly selected high power fields. The staining intensity was based on the average percentage of positive cells. The scoring results were analyzed by two blinded observers.

### Statistical analysis

The sample size in each experiment was chosen to ensure adequate power to detect a pre-specified effect. The results of at least three independent experiments were quantified by blinded observers and expressed as the mean ± SD (standard deviation). SPSS 22.0 statistical software package (SPSS Inc.) was used for statistical analysis. A two-tailed Student’s *t* test was used to evaluate the statistical differences between groups. Data meet normal distribution and *P* < 0.05 was considered statistically significant.

## Results

### Circ_0058106 was upregulated in HSCC tissues

**Fig. 1:**
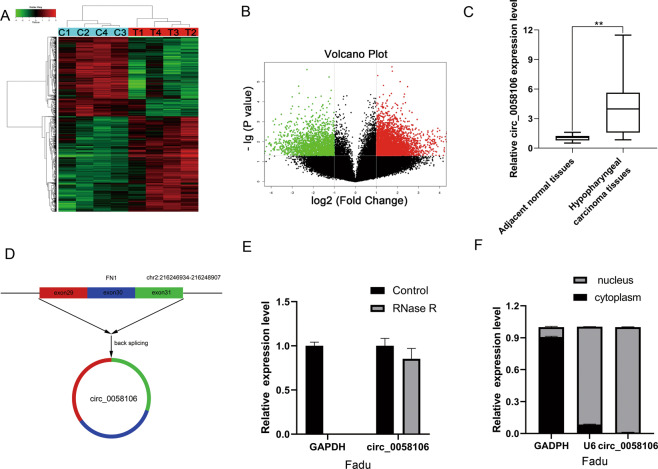
circ_0058106 was upregulated in HSCC tissues by using RNA profiling of circRNAs and qRT-PCR. **A** Heat map and hierarchical clustering analysis revealed different circRNA expression profiles between HSCC tissues (C1, C2, C3, and C4) and adjacent normal tissues (T1, T2, T3, and T4). **B** The volcano map showed that the circRNAs in HSCC tissues were dysregulated (red or green dots represent the upregulation or down-regulation of circRNA) in HSCC tissues. (FC ≥ 2.0, *P* value < 0.05) **C** circ_0058106 expression levels were significantly upregulated in tumor tissues than in corresponding normal tissues by using qRT-PCR. (*n* = 20, *P < 0.05; **P < 0.01) **D** The schematic diagram showed the head-to-tail splice site of the circ_0058106 PCR product amplified by divergent primers, and the splice site had been verified by Sanger sequencing. **E** Total RNA with or without RNase R (3U/mg) were treated for reverse transcription, circ_0058106 and GAPDH mRNA expression levels were detected by qRT-PCR. **F** qRT-PCR analysis indicated the distribution of GAPDH, U6 and circ_0058106 in the nucleus and cytoplasm of Fadu cells (*n* = 3).

Our lab performed microarray of hypopharyngeal cancer tissues in 2017 (Gene expression profiles, GSE 97418) [12]. CircRNA microarray was performed in the four paired HSCC tumor and adjacent normal tissues. Hierarchical cluster analysis clearly showed the difference in circRNA expression between the tumor group and the non-tumor group (Fig. 1A). A scatter plot was used to visualize the difference in circRNA expression between HSCC tumor tissues and paired normal tissues (Fig. 1B). The red dots and the green dots indicate the upregulation and down-regulation of circRNA with a fold change≥2.0 in HSCC tissue respectively. Circ_0058106 showed significantly increased expression levels in HSCC tissues compared with normal tissues. According to chip results we performed qRT-PCR in 20 pairs of HSCC tissues and adjacent normal tissues. Compared with the corresponding normal tissues, the expression levels of circ_0058106 in tumor tissues were significantly upregulated (Fig. 1C). Circ_0058106 consists of exon 29 to exon 31 of FN1 and is located on chromosome 2q33.1 (Fig. 1D). We then designed divergent primers and convergent primers of circ_0058106 and performed PCR for both cDNA and gDNA of Fadu cells (Fig. S1). The PCR amplification product of divergent primers of circ_0058106 was resistant to RNase R digestion (Fig. 1E). These results verified the cyclic structure of circ_0058106. By analyzing the relative expression of circ_0058106 in the cytoplasm and nucleus, we verified that circ_0058106 is mainly located in the nucleus of Fadu cells (Fig. 1F).

### Circ_0058106 overexpression promotes proliferation, migration, and invasion of HSCC cells in vitro

**Fig. 2:**
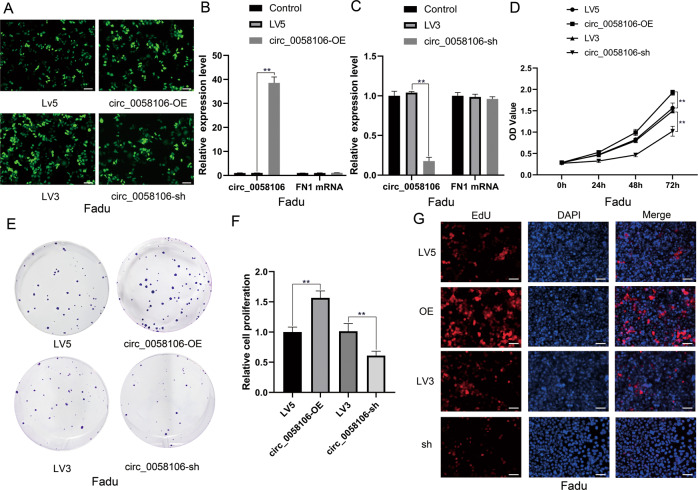
circ_0058106 overexpression promotes proliferation of HSCC cells in vitro. **A** GFP-tagged lentivirus in Fadu cells was observed by fluorescence microscope. (Scale bars = 100 μm) **B** Circ_0058106 and parental gene FN1 mRNA expression levels in circ_0058106 stably transfected and LV5 control Fadu cells were detected by qRT-PCR. (*n* = 3, *P < 0.05; **P < 0.01) **C** Circ_0058106 and parental gene FN1 mRNA expression levels in circ_0058106 stably knockdown and LV3 control Fadu cells were detected by qRT-PCR. (*n* = 3, *P < 0.05; **P < 0.01) **D** Proliferation of circ_0058106-OE, LV5, circ_0058106-sh and LV3 Fadu cells was measured by CCK8 assay at 0, 24, 48, and 72 h. (*n* = 3, *P < 0.05; **P < 0.01) **E** Proliferation of circ_0058106-OE, LV5, circ_0058106-sh and LV3 Fadu cells was measured by colon formation assay. **F** Quantitative analysis of colon formation assay. (*n* = 3, *P < 0.05; **P < 0.01) **G** Proliferation of circ_0058106-OE, LV5, circ_0058106-sh and LV3 Fadu cells was measured by EdU assay. (Scale bars = 100 μm).

In order to study the function of circ_0058106 in HSCC, we established circ_0058106 stably overexpressed (circ_0058106-OE) and stably knockdown (circ_0058106-sh) Fadu cell lines, and their respective negative controls LV5 and LV3. Fluorescent signal verified that cells had been successfully transfected with lentivirus (Fig. 2A). The overexpression and knockdown efficiency of circ_0058106 in Fadu cells was analyzed by qRT-PCR, the results showed that the expression levels of circ_0058106 but not the parental gene FN1 changed (Fig. 2B, C). We used CCK-8 cell viability analysis (Fig. 2D), colon formation analysis (Fig. 2E, F) and EdU analysis (Fig. 2G) to study the role of circ_0058106 in the proliferation of Fadu cells. The results showed circ_0058106 overexpression promoted cell proliferation, while knockdown of circ_0058106 inhibited proliferation in Fadu cells compared with the LV5 or LV3 control group, respectively.

**Fig. 3:**
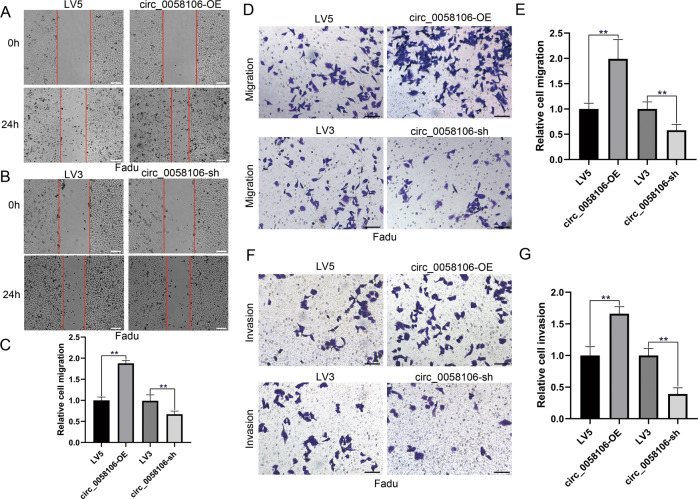
circ_0058106 overexpression promotes migration and invasion of HSCC cells in vitro. **A** Migration of circ_0058106-OE and LV5 was measured by wound healing assay. (Scale bars = 200 μm) **B** Migration of circ_0058106-sh and LV3 was measured by wound healing assay. (Scale bars = 200 μm) **C** Quantitative analysis of wound healing assay. (*n* = 3, *P < 0.05; **P < 0.01) **D** Migration of circ_0058106-OE, LV5, circ_0058106-sh and LV3 Fadu cells was measured by transwell migration assay. (Scale bars = 100 μm) **E** Quantitative analysis of transwell migration assay. (*n* = 3, *P < 0.05; **P < 0.01) **F** Invasion of circ_0058106-OE, LV5, circ_0058106-sh and LV3 Fadu cells was measured by transwell invasion assay. (Scale bars = 100 μm) **G** Quantitative analysis of transwell invasion assay. (*n* = 3, *P < 0.05; **P < 0.01).

We also used wound healing assay (Fig. 3A–C), transwell migration assay (Fig. 3D, E) and transwell invasion assay (Fig. 3F, G) to study the role of circ_0058106 in migration and invasion of Fadu cells. The results showed circ_0058106 overexpression enhanced cell migration and invasion ability, while knockdown of circ_0058106 decreased cell migration and invasion ability in Fadu cells compared with LV5 or LV3 control group, respectively. These results together indicated that circ_0058106 could promote Fadu cells proliferation, migration and invasion. Moreover, circ_0058106 also promoted human laryngeal carcinoma cells TU212 tumorigenesis and metastasis in vitro (Figs. S2, S3).

### Circ_0058106 overexpression promotes HSCC cells tumorigenesis and metastasis in vivo

**Fig. 4:**
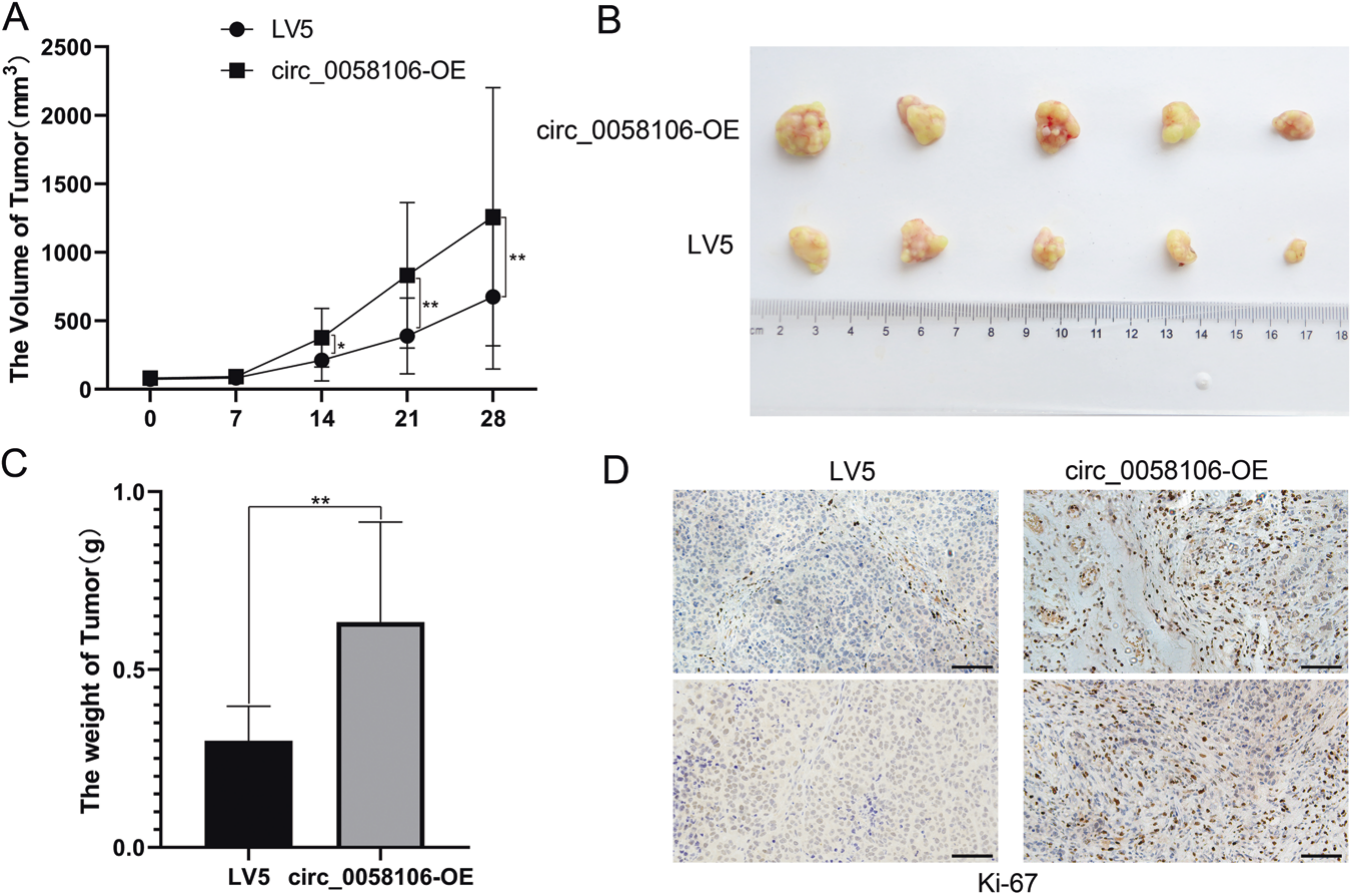
circ_0058106 overexpression promotes HSCC cells tumorigenesis in vivo. **A** circ_0058106-OE or LV5 Fadu cells were subcutaneously injected into nude mice (cell numbers = 5 × 10^6^, *n* = 5 for each group). Tumor size was monitored and calculated by caliper for up to 4 weeks (see Methods). The results were calculated as mean values ± SD. (*n* = 5, *P < 0.05; **P < 0.01) **B** A photo of five tumors aligned together were presented. **C** Tumor weight in circ_0058106-OE and LV5 group was measured. (*n* = 5, *P < 0.05; **P < 0.01) **D** Proliferation of circ_0058106-OE and LV5 group was measured by Ki-67 IHC strain. (Scale bars = 100 μm).

We then explored the role of circ_0058106 in HSCC in vivo by using nude mice transplanted tumor model. To confirm the biologic effect of circ_0058106 on HSCC cells growth in vivo, circ_0058106-OE and LV5 control Fadu cells were subcutaneously inoculated into nude mice respectively. The tumors formed by circ_0058106-OE Fadu cells grew faster than the control cells after inoculation (Fig. 4A). After 4 weeks, the mice were sacrificed. Compared with the control group, the size and weight of the tumors of the circ_0058106-OE group were also increased (Fig. 4B, C). The proportion of Ki67 positive cells in tumors verified the above results (Fig. 4D). These results demonstrated circ_0058106 could promote hypopharyngeal cancer growth in vivo.

**Fig. 5:**
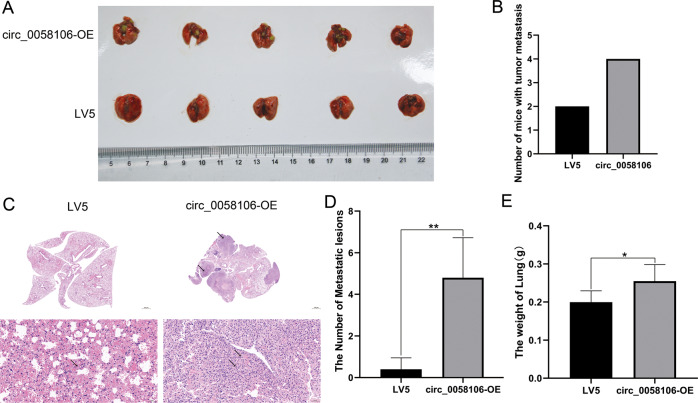
circ_0058106 overexpression promotes HSCC cells metastasis in vivo. **A** circ_0058106-OE or LV5 Fadu cells were subcutaneously tail vein injected into nude mice (cell numbers = 5 × 10^5^, *n* = 5 for each group). After 6 weeks the mice were sacrificed and a photo of five lung tissues aligned together was presented. **B** The number of mice with distant metastasis in circ_0058106-OE or LV5 group was measured. (*n* = 5, *P < 0.05; **P < 0.01) **C** Lung tissues were harvested from nude mice for HE stain and quantitated (Upper Scale bars = 1000 μm, lower scale bars = 50μm). **D** The number of metastatic lesions in circ_0058106-OE or LV5 group was measured. (*n* = 5, *P < 0.05; **P < 0.01) **E** The weight of lung tissues in circ_0058106-OE or LV5 group was measured. (*n* = 5, *P < 0.05; **P < 0.01).

We further studied the effect of circ_0058106 on the metastasis of hypopharyngeal carcinoma by using nude mouse metastasis model. The same number of circ_0058106-OE and control Fadu cells were injected into the lateral tail vein of nude mice aged 4–5 weeks. After 6 weeks, the mice were sacrificed. More nude mice with metastasis in lung were found in circ_0058106-OE group than LV5 control group (Fig. 5A, B). HE stain results showed more metastasis foci in the circ_0058106-OE group than in the LV5 control group (Fig. 5C, D), increased weights of lungs were also observed in the circ_0058106-OE group (Fig. 5E). These results demonstrated that circ_0058106 could promote hypopharyngeal cancer metastasis in vivo.

### Circ_0058106 directly binds miR-185-3p and regulates miR-185-3p expression in HSCC cells

**Fig. 6:**
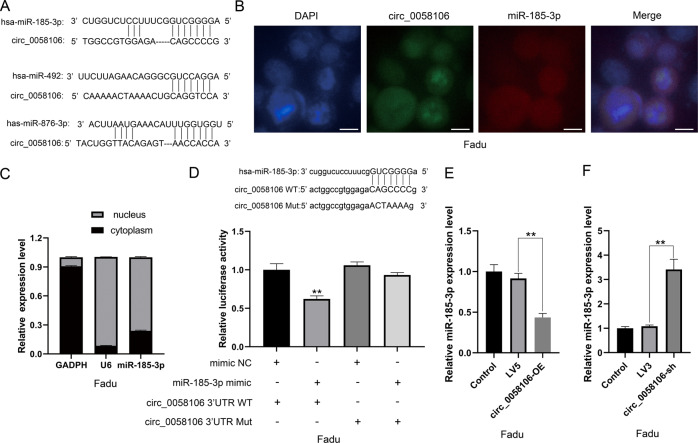
circ_0058106 binds miR-185-3p and regulates miR-185-3p expression in HSCC cells. **A** Sequence alignments between circ_0058106 and seed sequences of miRNAs. **B** FISH for subcellular localization of circ_0058106 and miR-185-3p by using 5′-CY3 labeled circ_0058106 FISH probe and 5′-FAM labeled miR-185-3p FISH probe, DAPI stained cell nuclei. (Scale bars = 10 μm) **C** qRT-PCR analysis indicated the distribution of GAPDH, U6 and miR-185-3p in the nucleus and cytoplasm of Fadu cells (*n* = 3). **D** Dual-luciferase reporter gene assay was used to detect interaction between circ_0058106 and miR-185-3p. (*n* = 3, *P < 0.05; **P < 0.01) **E** miR-185-3p expression in circ_0058106-OE and LV5 Fadu cells were detected by qRT-PCR. (*n* = 3, *P < 0.05; **P < 0.01) **F** miR-185-3p expression in circ_0058106-sh and LV3 Fadu cells were detected by qRT-PCR. (*n* = 3, *P < 0.05; **P < 0.01).

We further analyzed predicted miRNA targets and their interactions with circ_0058106 by using bioinformatics analysis. MiR-185-3p, miR-492 and miR-876-3p showed correlation with circ_0058106 (Fig. 6A). We chose miR-185-3p, which have been reported to be associated with nasopharyngeal progression for further study [13]. We then designed a specific circ_0058106 probe labeled with 5′-CY3 and a specific miR-185-3p probe labeled with 5′-FAM to detect their cellular distribution by using in situ hybridization. We verified that circ_0058106 and miR-185-3p are mainly located in the nucleus of Fadu cells (Fig. 6B). By analyzing the relative expression of miR-185-3p in the cytoplasm and nucleus, we also verified that miR-185-3p is mainly located in the nucleus of Fadu cells (Fig. 6C). We further constructed wild-type pmiRGlo-circ_0058106 vector containing miR-185-3p binding sites and mutant pmiRGlo-circ_0058106 vector for dual-luciferase reporter gene analysis, and co-transfected these vectors with miR-185-3p mimics to determine whether circ_0058106 can interact with miR-185-3p. The results showed that circ_0058106 directly bound to miR-185-3p (Fig. 6D). QRT-PCR results showed that the expression of miR-185-3p was decreased in circ_0058106-OE and increased in circ_0058106-sh Fadu cells compared with LV5 or LV3 control groups, respectively (Fig. 6E, F), indicating that circ_0058106 can negatively regulate miR-185-3p expression. Therefore, these results together suggested that circ_0058106 could directly bind miR-185-3p and negatively regulate the expression of miR-185-3p at the post-transcriptional level.

### Circ_0058106 promote EMT process by regulating Wnt2b/β-catenin/c-Myc pathway via miR-185-3p

**Fig. 7:**
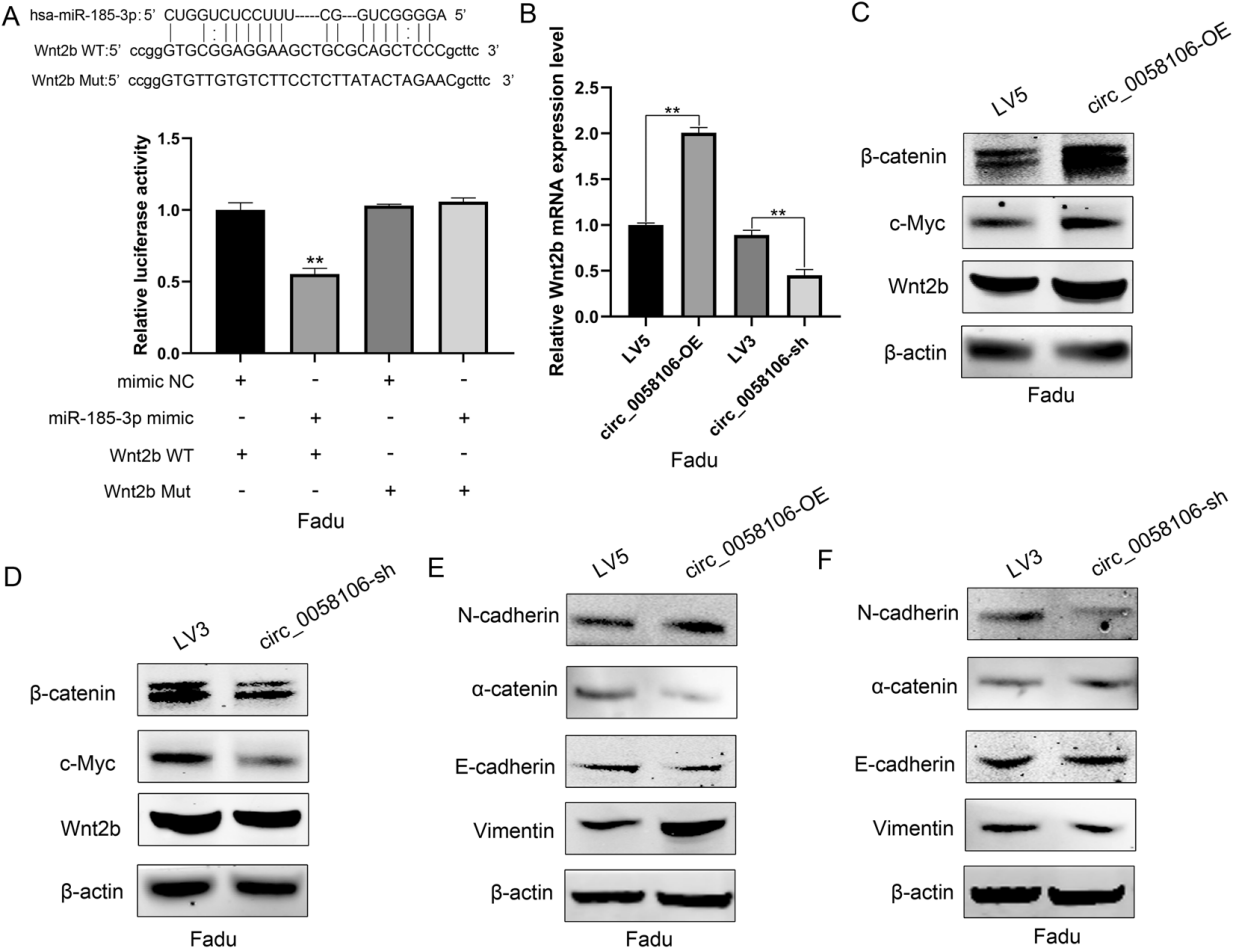
circ_0058106 promotes tumorigenesis and EMT process by regulating Wnt2b/β-catenin/c-Myc pathway via miR-185-3p in HSCC cells. **A** Dual-luciferase reporter gene assay was used to detect interaction between miR-185-3p and Wnt2b. (*n* = 3, *P < 0.05; **P < 0.01) **B** miR-185-3p expression in circ_0058106-OE, LV5, circ_0058106-sh and LV3 Fadu cells was detected by qRT-PCR. (*n* = 3, *P < 0.05; **P < 0.01) **C** Wnt2b, β-catenin and c-Myc protein expression in circ_0058106-OE and LV5 Fadu cells were detected by western blot. **D** Wnt2b, β-catenin and c-Myc protein expression in circ_0058106-sh and LV3 Fadu cells were detected by western blot. **E** N-cadherin, E-cadherin, Vimentin andα-catenin protein expression in circ_0058106-OE and LV5 Fadu cells were detected by western blot. **F** N-cadherin, E-cadherin, Vimentin and α-catenin protein expression in circ_0058106-sh and LV3 Fadu cells were detected by western blot.

Wnt2b/β-catenin/c-Myc signaling pathway plays an important role in tumorigenesis, tumor metastasis and the EMT process [14]. Previous studies have shown negative correlation between miR-185-3p and Wnt2b expression in nasopharyngeal cancer (NPC) cells and tissues. Luciferase reporter gene detection confirmed that miR-185-3p directly targets the coding region of Wnt2b [13]. miR-185-3p suppresses c-Myc protein level and activity in H1299 and HCT116 cells [15]. Since circ_0058106 binds miR-185-3p and regulates miR-185-3p expression, we speculated circ_0058106 may promote HSCC cells tumorigenesis, tumor metastasis and EMT process by regulating Wnt2b/β-catenin/c-Myc pathway via miR-185-3p. We constructed wild-type pmiRGlo-Wnt2b vector containing miR−185-3p binding sites and mutant pmiRGlo-Wnt2b vector for dual-luciferase reporter gene analysis, and co-transfected these vectors with miR-185-3p mimics to determine whether miR-185-3p can interact with Wnt2b. The results showed that miR-185-3p directly bound to Wnt2b (Fig. 7A). QRT-PCR results showed the expression of Wnt2b was increased in circ_0058106-OE and decreased in circ_0058106-sh cells compared with LV5 or LV3 control groups, respectively (Fig. 7B), indicating that circ_0058106 may regulate Wnt2b expression. Western blot results showed Wnt2b, β-catenin and c-Myc protein were increased in the circ_0058106-OE group and decreased in the circ_0058106-sh group compared with LV5 or LV3 control groups, respectively (Fig. 7C, D). Western blot of EMT markers showed epithelial marker E-cadherin and α-catenin were downregulated as well as mesenchymal marker N-cadherin and Vimentin were upregulated in circ_0058106-OE group compared with the LV5 control group (Fig. 7E). On the contrary, epithelial marker E-cadherin and α-catenin were upregulated as well as mesenchymal marker N-cadherin and Vimentin were downregulated in the circ_0058106-sh group compared with the LV3 control group. These results indicate circ_0058106 promotes HSCC cells tumorigenesis, tumor metastasis and EMT process by regulating Wnt2b/β-catenin/c-Myc pathway via miR-185-3p.

### MiR-185-3p reversed the effect of circ_0058106 in promoting tumorigenesis and metastasis of HSCC cells

**Fig. 8:**
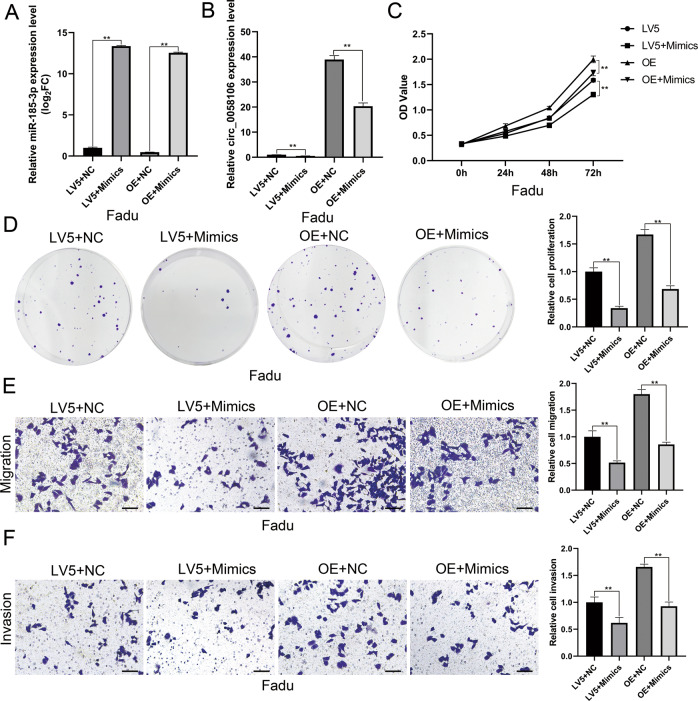
miR-185-3p mimics reverses the effect of circ_0058106 in HSCC cell in vitro. **A** miR-185-3p expression in circ_0058106-OE or LV5 Fadu cells after transfected with miR-185-3p mimics or negative control was measured by qRT-PCR. (*n* = 3, *P < 0.05; **P < 0.01) **B** circ_0058106 expression in circ_0058106-OE or LV5 Fadu cells after transfected with miR-185-3p mimics or negative control was measured by qRT-PCR. (*n* = 3, *P < 0.05; **P < 0.01) **C** Proliferation of circ_0058106-OE or LV5 Fadu cells after transfected with miR-185-3p mimics or negative control was measured by CCK8 assay at 0, 24, 48 and 72 h (*n* = 3, *P < 0.05; **P < 0.01) **D** Proliferation of circ_0058106-OE or LV5 Fadu cells after transfected with miR-185-3p mimics or negative control was measured by colony formation assay. (*n* = 3, *P < 0.05; **P < 0.01) **E** Migration of circ_0058106-OE or LV5 Fadu cells after transfected with miR-185-3p mimics or negative control was measured by transwell migration assay. (Scale bars = 100 μm, *n* = 3, *P < 0.05; **P < 0.01) **F** Invasion of circ_0058106-OE or LV5 Fadu cells after transfected with miR-185-3p mimics or negative control was measured by transwell invasion assay. (Scale bars = 100 μm, *n* = 3, *P < 0.05; **p < 0.01).

We then used miR-185-3p mimics to study whether miR-185-3p could reverse the effect of circ_0058106 on HSCC cells. QRT-PCR showed the expression level of circ_0058106 was downregulated both in LV5 and circ_0058106-OE cells after treated with miR-185-3p mimics (Fig. 8A, B). CCK-8 cell viability assay and colon formation assay showed miR-185-3p reversed the effect of circ_0058106 in promoting tumorigenesis of HSCC cells (Fig. 8C, D). Transwell migration assay and transwell invasion assay showed miR-185-3p reversed the effect of circ_0058106 in promoting metastasis and invasion of HSCC cells (Fig. 8E, F). To summary, miR-185-3p reversed the effect of circ_0058106 in promoting tumorigenesis, metastasis and invasion of HSCC cells.

## Discussion

Although circRNAs have been proved involved in tumorigenesis in many kinds of tumors, reports of circRNAs in hypopharyngeal cancer are seldom. Our lab conducted circRNA chip in four pairs of HSCC and adjacent normal tissue samples to study the role of circRNA in HSCC. Microarray data showed that 2392 circRNAs were significantly dysregulated in HSCC tissues, including 1304 upregulated and 1088 downregulated circRNA transcripts [12]. Chun et al. performed circRNA sequencing analysis on three pairs of tumors and adjacent normal samples from patients with hypopharyngeal cancer. The results showed that 173 circRNAs were dysregulated, including 71 upregulated and 102 downregulated circRNAs. Hsa_ circ_0008287 and hsa_circ_0005027 were proved to be downregulated in hypopharyngeal cancer and competitively bound with miR-548c-3p to control ErbB and Hippo signaling pathway genes [16]. However, research on the role of circRNA in hypopharyngeal cancer is still in its infancy.

**Fig. 9:**
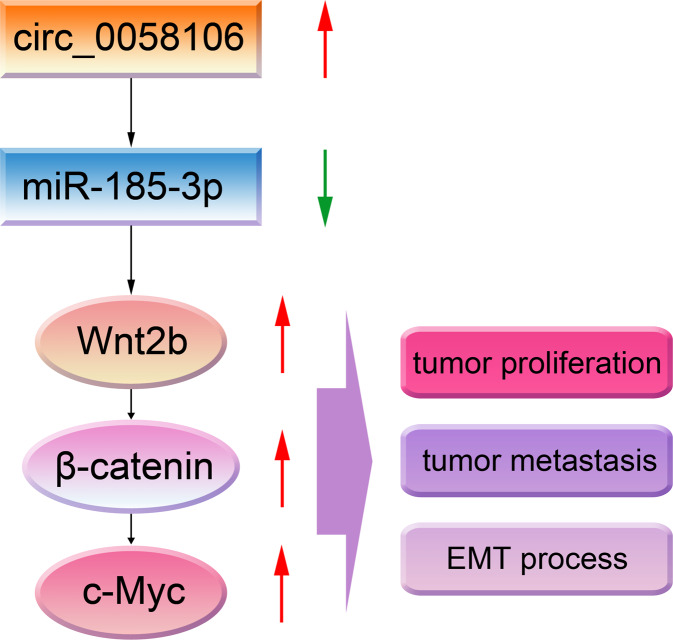
Diagram depicting the proposed role of circ_0058106 in HSCC. Circ_0058106 promotes proliferation, metastasis and EMT process by regulating Wnt2b/β-catenin/c-Myc pathway through miR-185-3p in HSCC.

In this study, we verified the upregulation of circ_0058106 in HSCC tissues compared with matched normal tissues, which is consistent with previous microarray results. We confirmed that circ_0058106 could promote HSCC cells proliferation, migration, invasion and EMT process by regulating Wnt2b/β-catenin/c-Myc pathway via sponging miR-185-3p (Fig. 9). Circ_0058106 is related to the progression and metastasis of HSCC. To sum up, we discovered a novel circRNA that promotes HSCC tumorigenesis and metastasis and studied related mechanisms.

MiR-185-3p was first identified in human neuroblastoma cells treated with retinoic [17]. MiR-185-3p is widely expressed in different tissues and cells of animals and plays important physiological regulation functions [15]. miR-185-3p has been proved as a tumor suppressor in a variety of tumors, including nasopharyngeal cancer [18], osteosarcoma [19], breast cancer [20], cervical cancer [21], prostate cancer [22], colorectal cancer [23], non-small cell lung cancer [24] and so on. FSCN1 [23], SMAD7 [18], YAP1 [19], E2F1 [20], BRD4 [24], FOXP3 [21] were confirmed as target proteins of miR-185-3p.

The Wnt family is a group of protein families related to many cell functions, such as organ formation, stem cell self-renewal, cell proliferation and apoptosis [25]. The Wnt/β-catenin pathway also called canonical Wnt pathway, which regulates the behavior of cells by regulating the LEF/TCF family DNA transcription, β-catenin which regulates transcriptional activity is a key member of the Wnt signaling pathway [26]. Downstream target genes of Wnt/β-catenin pathway include c-myc [27], Cyclin D1 [28], FGF family [29], MMP family [30], VEGF family [31] and so on. Wnt/β-catenin signaling pathway dysregulation is widespread in different types of human malignancies, including liver cancer [32], ovarian cancer [33], prostate cancer [34], head and neck cancer [35] and other malignancies. Wnt/β-catenin signaling pathway dysregulation could induce proliferation, survival, metastasis, EMT process and recurrence of tumors [36].

C-Myc is a proto-oncogene, which locates on human chromosome 8q24 and belongs to Myc family [37]. C-Myc regulates expression of many genes by regulating transcription process, including HIF-α [38], eIF4E [39], CDKN1A [40], GLUT1 [41], Bcl-2 [42] and so on. Therefore, c-Myc can regulate many biological functions in tumorigenesis, such as cell proliferation, apoptosis, metastasis, EMT process and stemness;[43] c-Myc dysregulation is widespread in different types of human malignancies, including breast cancer [44], glioma [45], liver cancer [46], gastric cancer [47], head and neck cancer [48] and other malignancies, suggesting that c-Myc may be a potential target for cancer treatment.

## Conclusion

In conclusion, our findings suggest that circ_0058106 promotes HSCC progression through miR-185-3p/Wnt2b/β-catenin/c-Myc signaling pathway. Therefore, circ_0058106 may be a potential therapeutic target and prognostic marker for HSCC treatment.

## Supplementary information

Supplementary legends

Figure S1

Figure S2

Figure S3

## Data Availability

The data that support the findings of this study are available from the corresponding author upon reasonable request.
