# Supplementary material for: Circ_0058106 promotes proliferation, metastasis and EMT process by regulating Wnt2b/β-catenin/c-Myc pathway through miR-185-3p in hypopharyngeal squamous cell carcinoma
Source: Cell Death Dis. 2021 Nov 9;12(11):1063. doi: 10.1038/s41419-021-04346-8 (PMC8575998; doi:10.1038/s41419-021-04346-8)
Supplement: Supplementary file 1 — Supplementary legends [file 41419_2021_4346_MOESM1_ESM.docx]

**Figure S1 PCR was performed for cDNA and gDNA of Fadu cells by using divergent primers or convergent primers of circ_0058106.**

**Figure S2 Circ_0058106 expression in four head and neck squamous cell carcinoma (HNSCC) cell lines were detected by qRT-PCR. (n = 3)**

**Figure S3 circ_0058106 promotes TU212 cells tumorigenesis and metastasis in vitro.**

**A** GFP-tagged lentivirus in TU212 cells was observed by fluorescence microscope. (Scale bars = 100μm) **B** Circ_0058106 and parental gene FN1 mRNA expression levels in circ_0058106 stably transfected and LV5 control as well as circ_0058106 stably knockdown and LV3 control TU212 cells were detected by qRT-PCR. (n = 3, *P < 0.05; **P < 0.01) **C D** Proliferation of circ_0058106-OE and LV5 control as well as circ_0058106-sh and LV3 control TU212 cells was measured by colon formation assay. (n = 3, *P < 0.05; **P < 0.01) **E F** Migration of circ_0058106-OE and LV5 control as well as circ_0058106-sh and LV3 control TU212 cells was measured by transwell migration assay. (Scale bars = 100μm，n = 3, *P < 0.05; **P < 0.01)
