# Supplementary figures and images for: Circ_0058106 promotes proliferation, metastasis and EMT process by regulating Wnt2b/β-catenin/c-Myc pathway through miR-185-3p in hypopharyngeal squamous cell carcinoma
Source: Cell Death Dis. 2021 Nov 9;12(11):1063. doi: 10.1038/s41419-021-04346-8 (PMC8575998; doi:10.1038/s41419-021-04346-8)

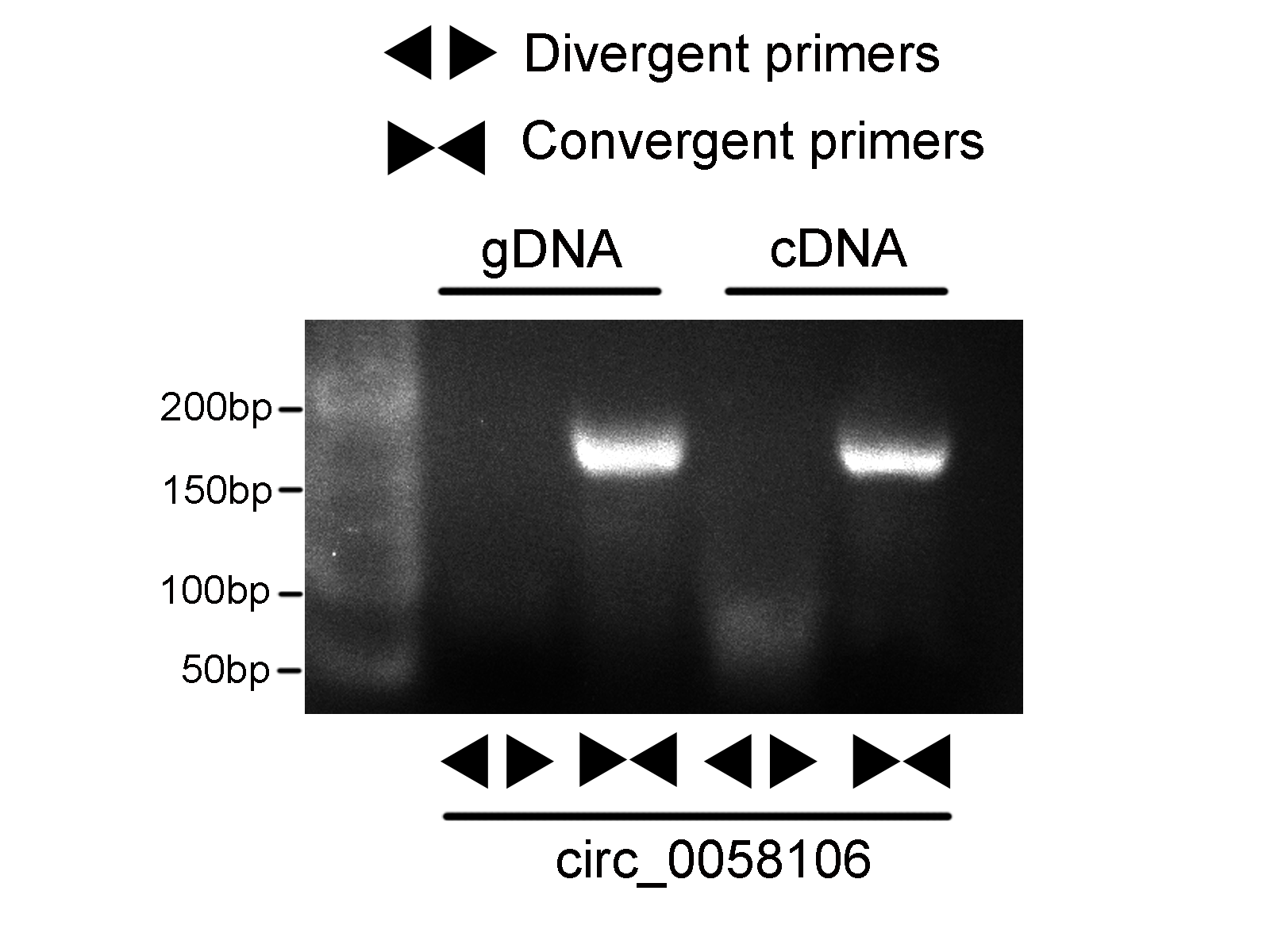

Supplement: Supplementary file 2 — Figure S1 [file 41419_2021_4346_MOESM2_ESM.tif]

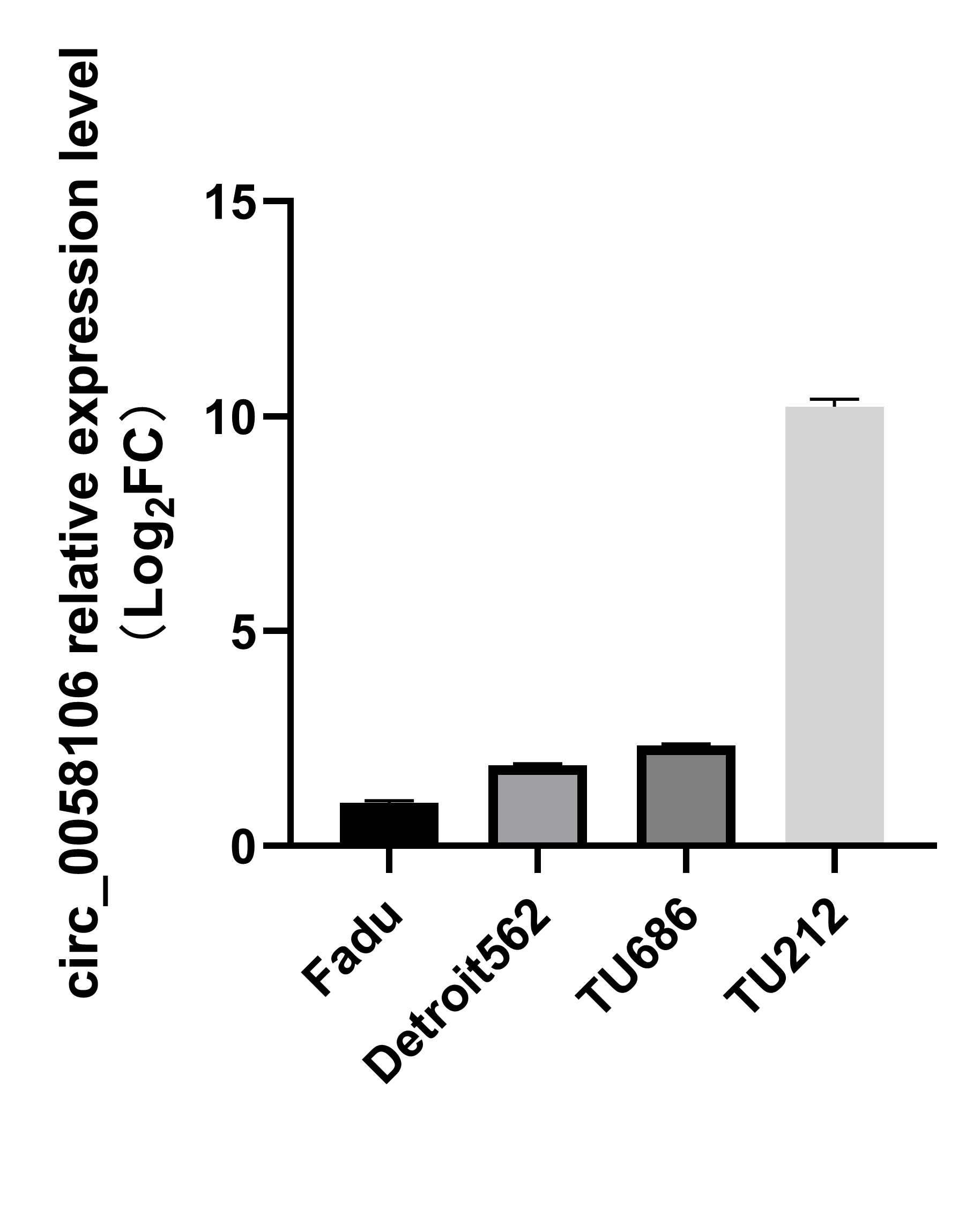

Supplement: Supplementary file 3 — Figure S2 [file 41419_2021_4346_MOESM3_ESM.tif]

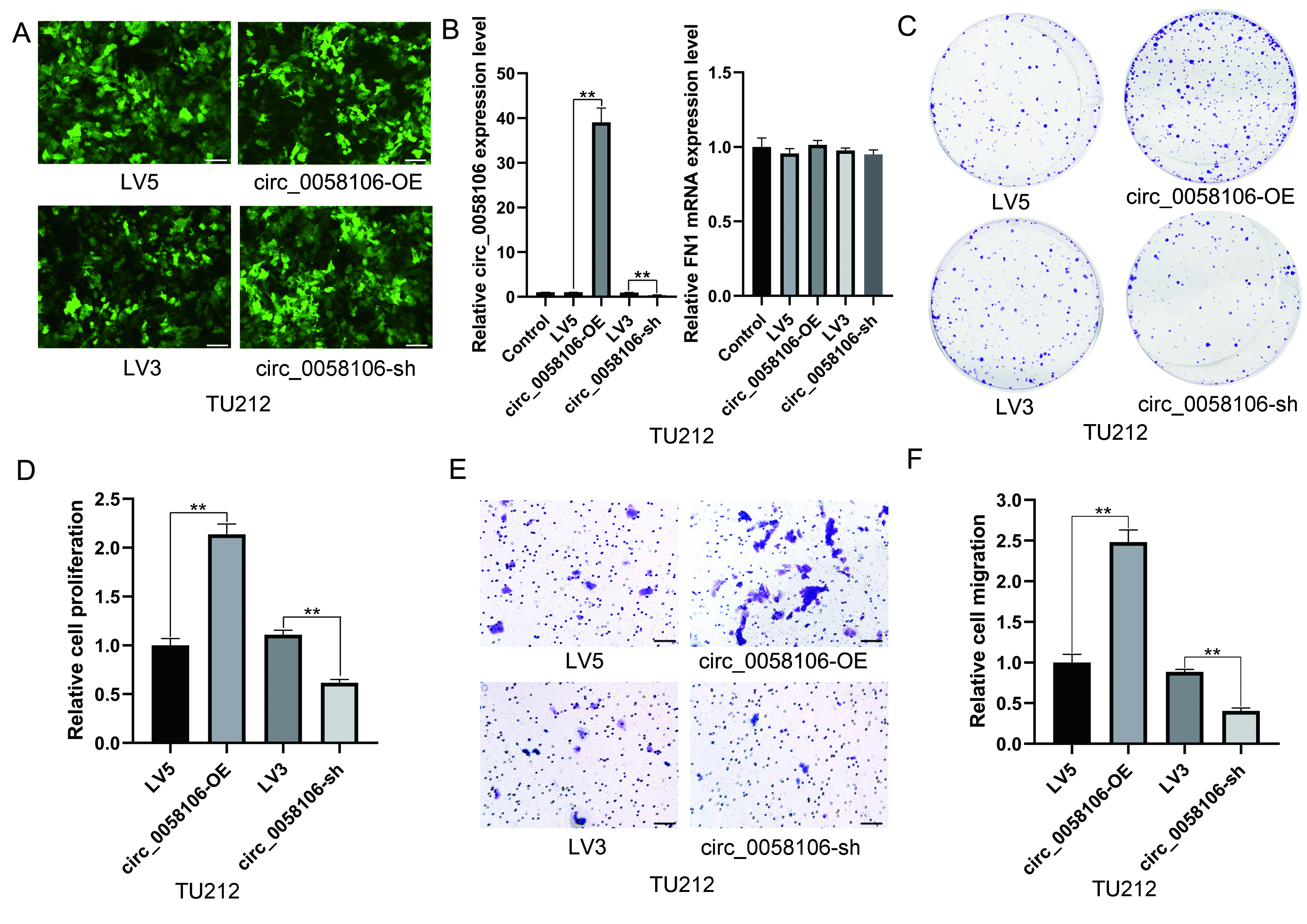

Supplement: Supplementary file 4 — Figure S3 [file 41419_2021_4346_MOESM4_ESM.tif]
